# Serological surveys to inform SARS-CoV-2 epidemic curve: a cross-sectional study from Odisha, India

**DOI:** 10.1038/s41598-021-89877-y

**Published:** 2021-05-18

**Authors:** Jaya Singh Kshatri, Debdutta Bhattacharya, Srikanta Kanungo, Sidhartha Giri, Subrata Kumar Palo, Debaprasad Parai, Jyotirmayee Turuk, Asit Mansingh, Hari Ram Choudhary, Matrujyoti Pattnaik, Girish Chandra Dash, Prasantajyoti Mohanty, Niranjan Mishra, Durga Madhab Satapathy, Sanjaya Kumar Sahoo, Sanghamitra Pati, ICMR-RMRC [OdiSHA-COVID-19] Serosurvey Team, Amiya Ranjan Mohanta, Anjan Kumar Bishoyee, Ashish Kumar Sadangi, Ashok Kumar Mahakuda, Biswakalyan Mishra, Dinabandhu Padhan, Gopinath Sethi, Hitesh kumar Jain, Janaki Biswal, Jeevan Kumar Mohanta, Jitendriya Amrit Pritam, Jwell Kiran Pradhan, Kanhu Charan sahoo, Keshab Chandra Dalai, Manas kumar Bhoi, Nirupama Sahoo, Nutan Dwibedi, Pradyuspita Sahoo, Sadruddin Khan, Sagarkanta Pradhan, Satyabrata Rout, Shakti Ranjan Barik, Sithun Kumar Patro, Smitanjali Samal, Soni Soni, Spandan Kumar Bhanjadeo, Srikant Kumar Patra, Subhralaxmi Dwivedy, Subrat kumar Nayak, Trilochan Bhoi

**Affiliations:** 1 ICMR-Regional Medical Research Centre, Bhubaneswar, India; 2 Department of Health and Family Welfare, Government of Odisha, Bhubaneswar, India; 3 Department of Community Medicine, Maharaja Krushna Chandra Gajapati Medical College and Hospital, Berhampur, India

**Keywords:** Epidemiology, Viral infection

## Abstract

This was a population based cross-sectional study carried out to estimate and compare the seroprevalence, hidden prevalence and determine the demographic risk factors associated with SARS-CoV-2 infection among adults in the three largest cities of Odisha, India, and ascertain the association with the progression of the epidemic. The survey carried out in August 2020 in the three largest cities of the state of Odisha, India. Blood samples were collected from the residents using random sampling methods and tested for anti- SARS CoV-2 antibodies using an automated CLIA platform. A total of 4146 participants from the 3 cities of Bhubaneswar (BBS), Berhampur (BAM) and Rourkela (RKL) participated. The female to male participation ratio was 5.9:10 across the three cities. The gender weighted seroprevalence across the three cities was 20.78% (95% CI 19.56–22.05%). While females reported a higher seroprevalence (22.8%) as compared to males (18.8%), there was no significant difference in seroprevalence across age groups. A majority of the seropositive participants were asymptomatic (90.49%). The case to infection ratio on the date of serosurvey was 1:6.6 in BBS, 1:61 in BAM and 1:29.8 in RKL. The study found a high seroprevalence against COVID-19 in urban Odisha as well as high numbers of asymptomatic infections. The epidemic curves had a correlation with the seroprevalence.

## Introduction

The COVID19 pandemic has so far affected 216 countries and caused more than 32 million cases and about a million deaths worldwide^1^. India has the second largest number of cases at 5.9 million and has been reporting about a quarter of the daily global incident cases for some time now^2^. While the pandemic is at different stages across the country, there seem to be significant local differences in the progression within the states as well. The state of Odisha in eastern India contributes over 3% of the active case load but with less than 1% of the cumulative mortality in the country^2^. The pandemic in the state has till now been largely driven by urban clusters with major cities contributing the most to the case load^3^. Large population size with high density, presence of slums, variable adherence to preventive measures, and a sizeable migrant population seem to be the common characteristics driving the transmission of infection in these cities. These regions remain critical to the COVID-19 response of the state.

As with any novel respiratory infection, there is an uncertainty of epidemiological, serological, infectivity, and virulence-related information of SARS-CoV-2^4^. Testing strategies and capacities have been evolving, but broadly, until now, it has been focused on the symptomatic and higher risk groups, which tend to overestimate the burden of the infection in the community due to a biased denominator^4–6^. Additionally, the role of pre-symptomatic, asymptomatic or subclinical infections in disease transmission dynamics is also not well understood^4^. Asymptomatic or subclinical individuals are those having SARS-CoV-2 infection but without any typical symptoms identified for COVID-19. Among these asymptomatic cases, those who develop symptoms in the later stage of infection are defined as pre-symptomatic. These two categories are directed either for home isolation or in a COVID care centre and need not any medical treatment if not serious.

While viral nucleic acid detection by real-time polymerase chain reaction (RT-qPCR) test from the nasopharyngeal swab is considered the gold standard frontline test for clinical diagnosis of SARS-CoV-2^7^. However, the positive case reporting couldn’t give a complete picture of the actual infected case numbers due to the limitations like high testing time, lower laboratory testing capacity, the requirement of highly skilled technicians, absence of typical symptoms. Usually, antibody tests can be used for disease detection after 5–7 days of illness. IgM antibodies are evident in the blood for the first two months and IgG antibodies generally start appearing after two weeks of the onset of infection and last for several months^8–10^. Thus, although these tests are not useful for detecting acute infection. Population-based sero-epidemiological studies could be useful to understand the cumulative exposure levels to the infection and make inferences on the actual burden of infection, its geographical spread, effect on specific demographic/risk groups, gaps in testing and infection fatality rates^4,10^. The information from such studies will also be helpful for monitoring the extent of the ongoing immunization programs as vaccination drives are already started in different countries including India^11^. This evidence will inform the policy makers to plan and implement public health interventions for the prevention and control of the pandemic. The state of Odisha has been supporting a multidisciplinary research effort, the “Odisha COVID-19 Study Group”, comprising of researchers, clinicians, and managers from over 10 institutions and UN bodies in the state to generate evidence to better inform the policy response to the ongoing pandemic. As part of this group, the Odisha Sero-surveillance and health assessment for COVID-19 (OdiSHA-COVID-19) study was proposed to assess the extent of infection in the community and specific groups with the following objectives:

## Objective

To estimate and compare the seroprevalence, hidden prevalence and determine the demographic risk factors associated with SARS-CoV-2 infection among adults in the three largest cities of Odisha, India, and ascertain the association with the progression of the epidemic.

## Methodology

This was a population-based cross-sectional serological survey carried out in August 2020 in the three largest cities of the state of Odisha in eastern India with a total population of over 2 million. The study population was randomly selected from the community members of the municipal wards from each of the cities. Adults residing in the city for at least the past 3 months and who agreed to provide written informed consent for data and sample collection were included in the study. We excluded pregnant women, bedridden patients, and those with recognizable cognitive impairment. Minimum sample size per city was calculated to be 1437. This was done on the Open Epi ver3.0 software using the following formula:

$$ {\text{n }} = \, \left[ {{\text{Deff}}*{\text{Np}}\left( {{1} - {\text{p}}} \right)} \right]/ \, [\left( {{\text{d2}}/{\text{Z21}} - \alpha /{2}*\left( {{\text{N}} - {1}} \right) + {\text{p}}*\left( {{1} - {\text{p}}} \right)} \right] $$

where, n = sample size, Deff = design effect, N = population size, p = estimated proportion, q = 1-p, d = desired precision or absolute level of precision. We assumed a seroprevalence of 15%, which has been reported in urban regions of India during the same period, relative precision of 20%, a design effect of 2.2 (calculated using a weak interclass correlation and a cluster size of 60), power of 80%, a finite population and a non-response rate of 20%^12–14^. This was rounded off to 1500 per city.

**Figure 1:**
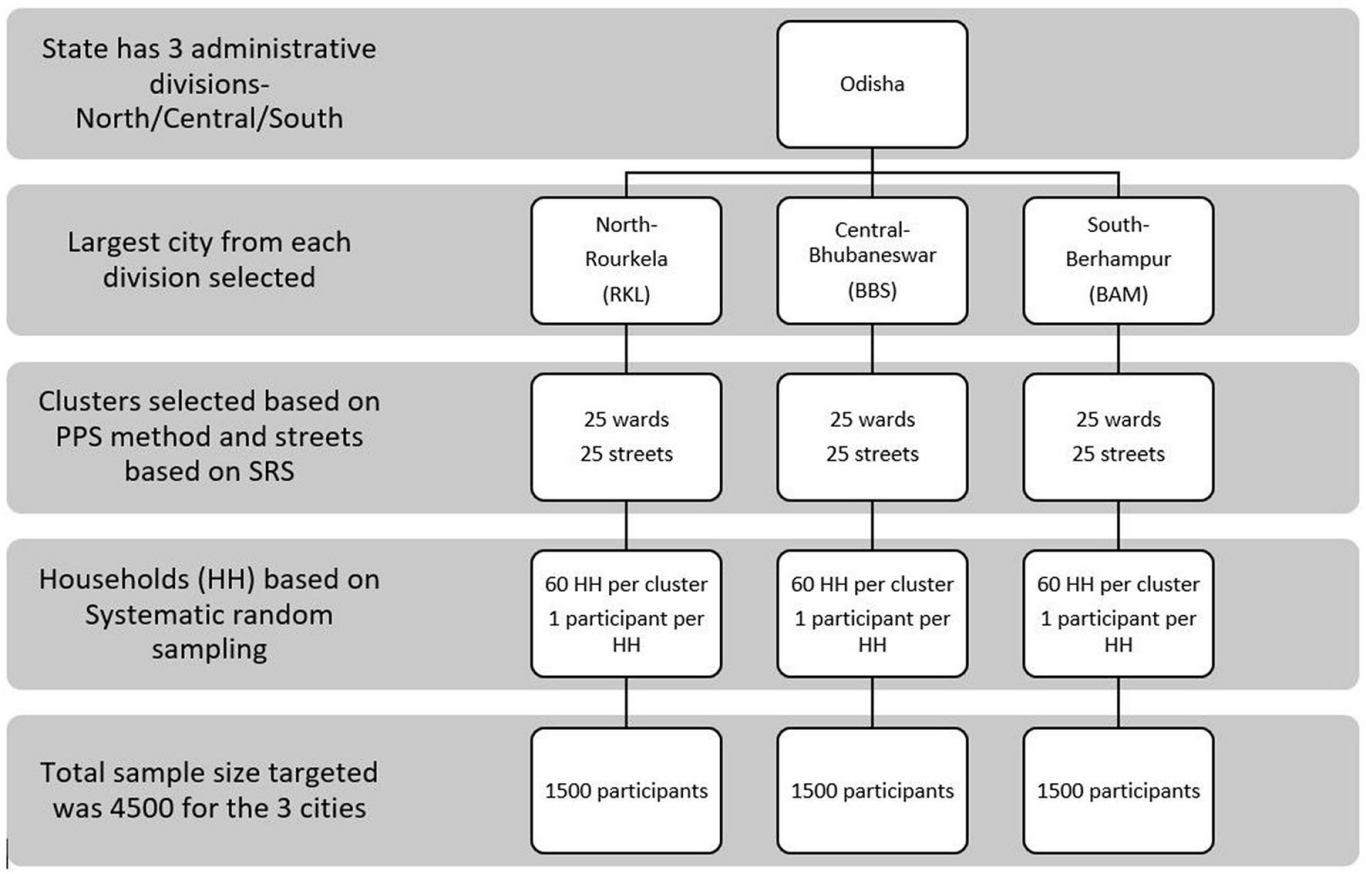
Sampling framework of the Multi-stage sampling design.

Multi-stage random sampling was used for recruiting participants. For every city, the municipal wards were treated as clusters and 25 wards were selected based on a probability proportional to size. Residential street names in the ward were listed and the street from where the sampling began (as well as the direction of sampling) in each cluster was selected by a computerized simple random method. Households in the street were selected using systematic random sampling and one eligible individual was selected from each household using an age-ordered matrix. Locked houses and/or nonresponse were recorded and the sampling frame was shifted from one household to the immediate adjacent house in these cases. The sampling framework is provided below in Fig. 1.

Data on socio-demographic variables, exposure history with a confirmed (and/or suspected) case, symptom profile in the last 30 days, geographical location, travel, and testing history were collected in a structured tool by trained field investigators who conducted participant interviews. An Open Data Kit based electronic data capture tool was used for this purpose. Following all aseptic precautions, 3–4 ml blood samples were collected in the field by trained phlebotomists by venepuncture and transferred to vacutainers. These were transported maintaining a cold chain (2–8° C) to the serology laboratory at the Indian Council of Medical Research-Regional Medical Research Centre in Bhubaneswar (ICMR-RMRC) for analysis. Additionally, secondary data on the daily number of antigen tests carried out, the number of positives and deaths due to COVID-19 were obtained for the past 3 months from government sources directly.

Serum samples were subjected to detection in Roche Cobas e411 for the presence of IgG antibodies against COVID-19 using Electro-chemiluminescence immunoassay (ECLIA) based technique which is based on the test principle of double-antigen sandwich assay and provides the result in 18 min. Elecsys® Anti-SARS-CoV-2 is an immunoassay for the in vitro qualitative detection of antibodies (including IgG) to SARS-CoV-2 in human serum and plasma. The assay uses a recombinant protein representing the nucleocapsid (N) antigen for the determination of antibodies against SARS-CoV-2. The test is intended as an aid in the determination of the immune reaction to SARS-CoV-2.

Testing procedures were followed as per the manufacturer’s instructions. Serum samples (20 μL) were incubated with a mix of biotinylated and ruthenylated nucleocapsid (N) antigens. Double-antigen sandwich immune complexes are formed in the presence of corresponding antibodies. After the addition of streptavidin-coated microparticles, the pre-formed complexes bind to the solid phase via the interaction of biotin and streptavidin. After that, the reagent mixture was transferred to the measuring cell, where the microparticles were magnetically captured onto the surface of the electrode. Unbound substances were subsequently removed. Electrochemiluminescence was then induced by applying a voltage and measured with a photomultiplier. The signal yield increased with the antibody titre. The value was expressed in Cut off Index (CoI) and a value of < 1.0 was considered nonreactive and COI ≥ 1.0 was reactive.

The seroprevalence of SARS-CoV-2 infection was estimated as a proportion along with 95% confidence intervals and its distribution assessed across cities and demographic parameters. Gender weights were added in prevalence estimates to account for a higher non-response rate in females. The infection-to-case ratio and the infection fatality rate were calculated. Median time of seroconversion was assessed by a time-dependent plot among those previously tested positive for SARS-CoV-2 by real-time polymerase chain reaction (RT-qPCR). Temporal comparison of the community seroprevalence estimates with the detected number of cumulative cases, active cases, recoveries, and deaths are done. Heat maps with varying seroprevalence were built for each of the city’s wards. Statistical analyses were done using R (ver. 4.0.2) software packages and GIS analysis was done using QGIS (ver. 3.10).

Interviews were conducted ensuring privacy. All data was stored securely under the investigator’s responsibility, with a focus on ensuring the confidentiality of study participants. The final report and publications are based on aggregate data without any identifying information. A database with electronic tracking, password-restricted access, and audit trails, with time and date stamps on data entry and edits, was used for quality control.

Approval for the protocol was obtained from the ICMR RMRC Institutional Human Ethics Committee and the State Health and Research Ethics Committee. All methods were performed in accordance with ICMR-National ethical guidelines for biomedical research involving human participants. The study methods, analyses, and reporting have been performed per the WHO Unity protocol and ICMR National Serosurvey protocol in India^15,16^.

## Results

**Figure 2:**
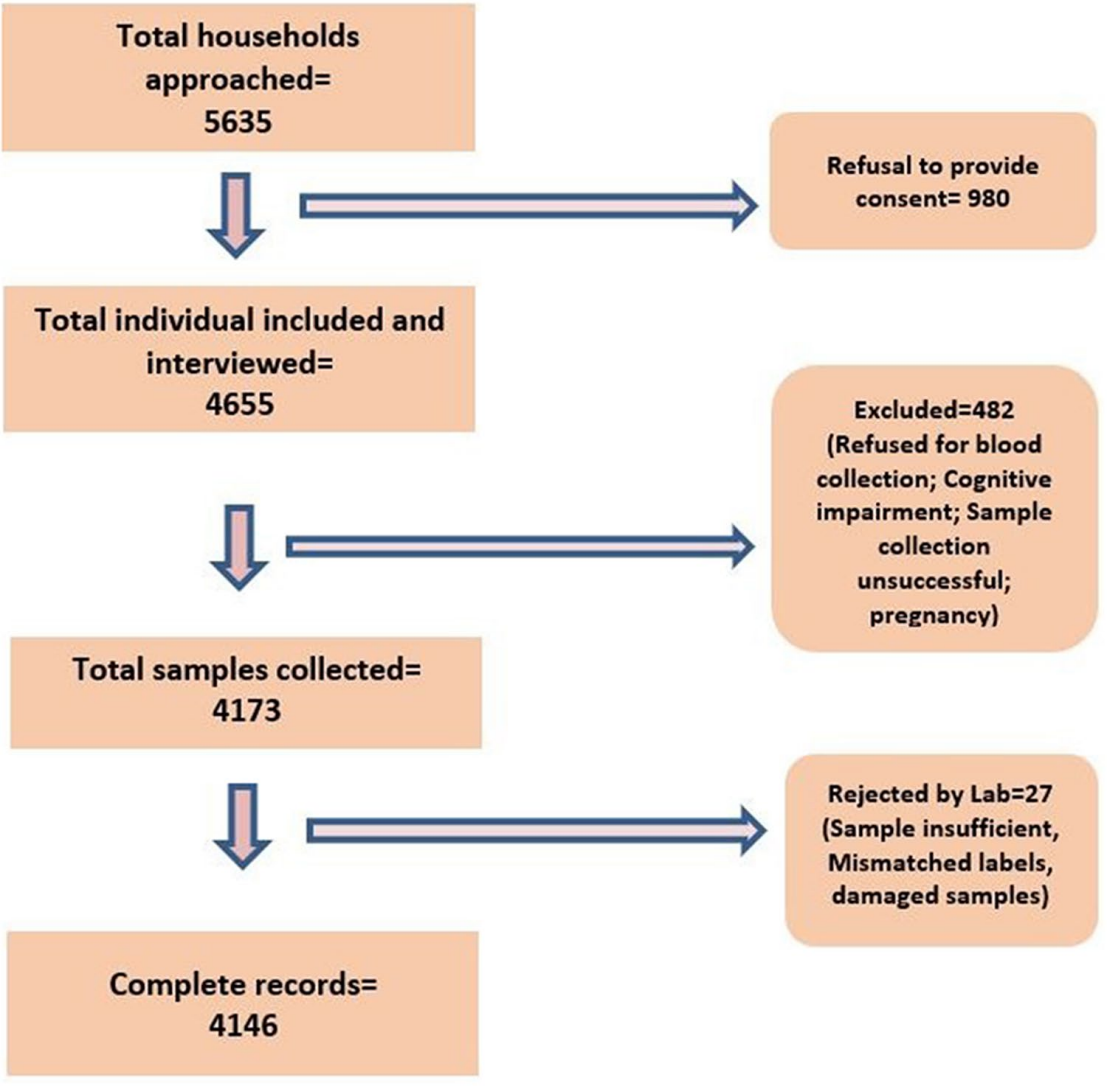
Study Flow diagram of the sample collection process.

The study was conducted among 4146 participants from the 3 cities of Bhubaneswar (BBS), Berhampur (BAM), and Rourkela (RKL). A total of 5635 households were approached and the average non-response rate in the community was 17.4% (980/5635), which was similar across the three cities. The study flow diagram is provided below in Fig. 2.

Among the study participants, 1553 were females and the rest males. The non-response rates were significantly higher among females (27.6%; 583/2111) as compared to males (12.4%; 437/3524). The mean age of the study participants was 44.20 (± 14.2) years.

The gender-weighted seroprevalence across the three cities was 20.78% (861.53/4146; 95% CI 19.56–22.05%). This was highest in BAM at 31.14% (428.17/1375; 95% CI 28.69–33.66%) followed by 24.59% (357.05/1452; 95% CI 22.39–26.88%) in RKL and 5.24% (69.12/1319; 95% CI 4.10–6.58%) in BBS. While females reported a higher seroprevalence (22.79%; 354/1553) as compared to males (18.81%; 488/2593), there was no significant difference in seroprevalence across age groups.

The demographic characteristics of the study population and the distribution of seroprevalence are provided in Table 1.

**Table 1:** Demographic characteristics of the study population and the distribution of seroprevalence.

Characteristic		City						Total		χ^2^ (p-value)
		BBS		BAM		RKL				
		N =	Positive = (%, 95% CI)	N =	Positive = (%, 95% CI)	N =	Positive = (%, 95% CI)	N =	Positive = (%, 95% CI)	
Age group	Less than 20 years	47	04 (8.51%, 2.36–20.37%)	31	08 (25.80%, 11.85–44.61%)	39	08 (20.51%, 9.29–36.46%)	117	20 (17.09%, 10.76–25.15%)	8.483 (.132)
	20–29 years	229	12 (5.24%, 2.73–8.97%)	146	49 (33.56%, 25.96–41.83%)	217	50 (23.04%, 17.61–29.22%)	592	111 (18.75%, 15.68–22.13%)	
	30–39 years	298	23 (7.71%, 4.95–11.35%)	251	90 (35.85%, 29.92–42.12%)	344	82 (23.83%, 19.43–28.69%)	893	195 (21.83%, 19.16–24.69%)	
	40–49 years	311	08 (2.57%, 1.11–5.00%)	364	114 (31.31%, 26.58–36.35%)	351	80 (22.79%, 18.50–27.54%)	1026	202 (19.68%, 17.29–22.25%)	
	50–59 years	258	09 (3.48%, 1.60–6.51%)	271	77 (28.41%, 23.1234.18%)	287	65 (22.64%, 17.93–27.93%)	816	151 (18.50%, 15.89–21.34%)	
	≥ 60 years	176	13 (7.38%,3.99–12.29%)	312	89 (28.52%, 23.58–33.88%)	214	61 (28.50%, 22.22–35.05%)	702	163 (23.21%, 20.14–26.52%)	
Gender	Male	891	46 (5.16%, 3.80–6.82%)	847	260 (30.69%, 27.60–33.92%)	855	182 (21.28%, 18.58–24.18%)	2593	488 (18.81%, 17.33–20.37%)	9.481 (< 0.01)
	Female	428	23 (5.37%, 3.43–7.95%)	528	167 (31.62%, 24.40–31.76%)	597	164 (27.47%, 23.92–31.24%)	1553	354 (22.79%, 20.73–24.96%)	
Occupation	Manufacturing	62	6 (9.67%, 3.63–19.88%)	120	47 (39.16%, 30.38–48.49%)	66	17 (25.75%, 15.77–38.00%)	248	70 (28.22%, 22.71–34.26%)	62.565 (< 0.01)
	Public service	200	11 (5.5%, 2.77–9.62%)	133	34 (25.56%, 18.40–33.84%)	267	44 (16.47%, 12.23–21.48%)	600	89 (14.83%, 12.08–17.93%)	
	Private service	295	7 (2.37%, 0.96–4.83%)	161	41 (25.46%, 18.93–32.92%)	226	40 (17.69%, 12.95–23.31%)	682	88 (12.90%, 10.48–15.65%)	
	Self employed	206	11 (5.33%, 2.69–9.35%)	295	105 (35.59%, 30.12–41.34%)	161	50 (31.05%, 24.00–38.81%)	662	166 (25.07%, 21.81–28.55%)	
	Homemaker	251	10 (3.98%, 1.92–7.20%)	303	90 (29.70%, 24.61–35.19%)	377	112 (29.70%, 25.13–34.60%)	931	212 (22.77%, 20.11–25.60%)	
	Student	73	3 (4.10%, 0.85–11.74%)	58	18 (31.03%, 19.53–44.54%)	76	16 (21.05%, 12.53–31.92%)	207	37 (17.87%, 12.90–23.78%)	
	Unemployed	101	8 (7.92%, 3.48–15.01%)	144	38 (26.38%, 19.40–34.37%)	129	39 (30.23%, 22.46–38.93%)	374	85 (22.72%, 18.57–27.31%)	
	Others/retired	131	13 (9.92%, 5.39–16.37%)	161	54 (33.54%, 26.30–41.39%)	150	28 (18.66%, 12.77–25.83%)	442	95 (21.49%, 17.75–25.62%)	
Household Size	≤ 2	239	10 (4.18%, 2.02–7.55%)	228	50 (21.92%, 16.73–27.86%)	185	38 (20.54%, 14.96–27.08%)	652	98 (15.03%, 12.37–18.00%)	37.457 (< 0.01)
	3–4	674	32 (4.74%, 3.26–6.63%)	669	197 (29.44%, 26.01–33.06%)	722	155 (21.46%, 18.52–14.64%)	2065	384 (18.59%, 16.93–20.34%)	
	5–6	307	18 (5.86%, 3.51–9.10%)	339	129 (38.05%, 32.86–43.45%)	375	102 (27.20%, 22.75–32.00%)	1021	249 (24.38%, 21.78–27.14%)	
	> 6	99	9 (9.09%, 4.24–16.55%)	139	51 (36.69%, 28.68–45.27%)	170	51 (30.00%, 23.22–37.49%)	408	111 (27.20%, 22.94–31.08%)	
Travel history	Yes	74	1 (1.35%, 0.03–7.30%)	22	6 (27.27%, 10.72–50.22%)	26	4 (15.38%, 4.35–34.86%)	122	11 (9.01%, 4.58–15.56%)	9.904 (< 0.01)
	No	1245	68 (5.46%, 4.26–6.87%)	1353	421 (31.11%, 28.65–33.65%)	1426	342 (23.98%, 21.78–26.28%)	4024	831 (20.65%, 19.40–21.93%)	
Total		1319	69 (5.23%; 4.09–6.47%)	1375	427 (31.05%; 28.61–33.57%)	1452	346 (23.82%; 21.62–26.10%)	4146	842 (20.30%; 19.0–21.56%)	–

Among the study population, 6.12% (254/4146) had developed symptoms suggestive of COVID-19 in the past 30 days and 9.98% (414/4146) had been tested by RT-qPCR for COVID-19. A majority of the seropositive participants were asymptomatic (90.49%; 762/842). Among those who reported symptoms, the most common symptom was fever (68.89%; 175/254), followed by cough (46.06%; 117/254) and myalgia (32.67%; 109/254)). The distribution of seroprevalence according to symptoms and testing is given below in Table 2.

**Table 2:** Testing status and symptom profile of the study participants.

Testing/symptom profile		N =	Seropositive (%, 95% CI)	χ^2^ (p-value)
Nasopharyngeal swab test by RT-qPCR	Yes	414	90 (21.73%, 17.85–26.02%)	0.581 (0.446)
	No	3732	752 (20.15%, 18.87–21.47%)	
RT-qPCR result	Positive	29	23 (79.31%, 60.3%-92.0%)	60.752 (< 0.01)
	Negative	385	67 (17.40%, 13.7%-21.6%)	
Symptoms of self (last 30 days)	Yes	254	80 (31.49%, 25.83–37.59%)	20.924 (< 0.01)
	No	3892	762 (19.57%, 18.34–20.86%)	
Symptoms in household members (last 30 days)	Yes	51	11 (21.56%, 11.29–35.32%)	0.051 (0.822)
	No	4095	831 (20.29%, 19.07–21.55%)	
Symptom profile	Fever	175	64 (36.57%, 29.44–44.17%)	29.85 (< 0.01)
	Cough	117	37 (31.62%, 23.33–40.86%)	9.525 (< 0.01)
	Shortness of breath/ difficulty in breathing	74	12 (16.21%, 8.66–26.61%)	.586 (0.444)
	Myalgia	109	28 (25.68%, 17.79–34.94%)	2.002 (0.157)
	Headache	83	24 (28.91%, 19.48–39.90%)	3.877 (0.049)
	Diarrhea	12	7 (58.33%, 27.66–84.83%)	10.752 (< 0.01)
	Sore throat	34	12 (35.29%, 19.74–53.51%)	4.75 (0.029)
	Anosmia/loss of taste	12	3 (25%, 5.48–57.18%)	0.164 (0.686)

Among those who had seroconversion and had been tested positive by RT-qPCR, the median duration between both was 31 days.

**Figure 3:**
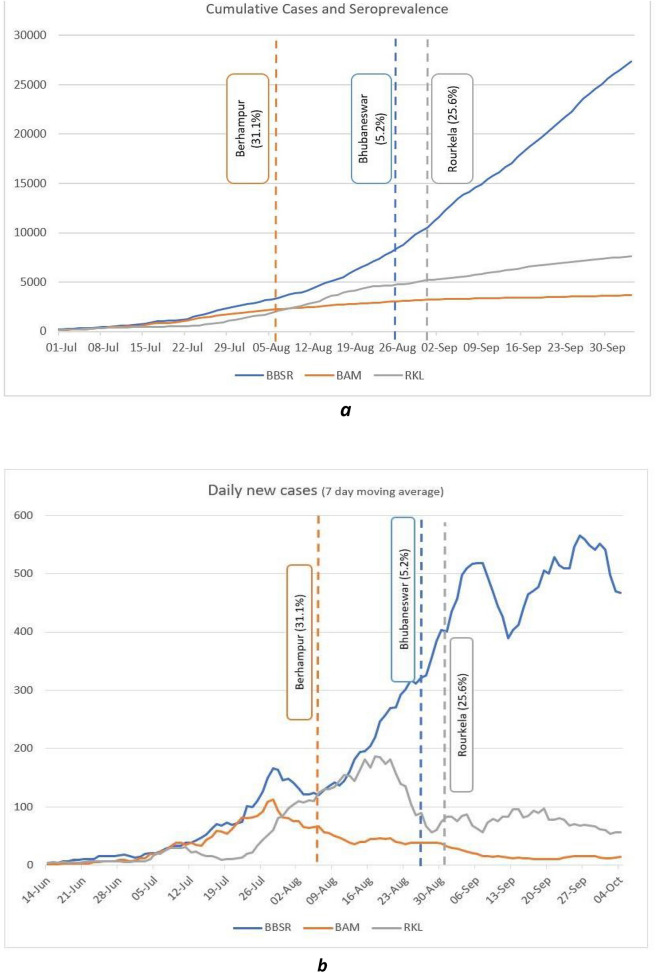
(**a**) 7 days moving average of new cases detected in three cities and point of seroprevalence. (**b**) Cumulative cases detected in the three cities and point of seroprevalence estimates.

The cumulative number of cases detected on 1st September was 11,641 in BBS, 3277 in BAM, and 5362 in RKL. The association between time trends of the progression of the daily new cases and cumulative cases and the time point of seroprevalence estimates is given in Fig. 3a,b below.

**Figure 4:**
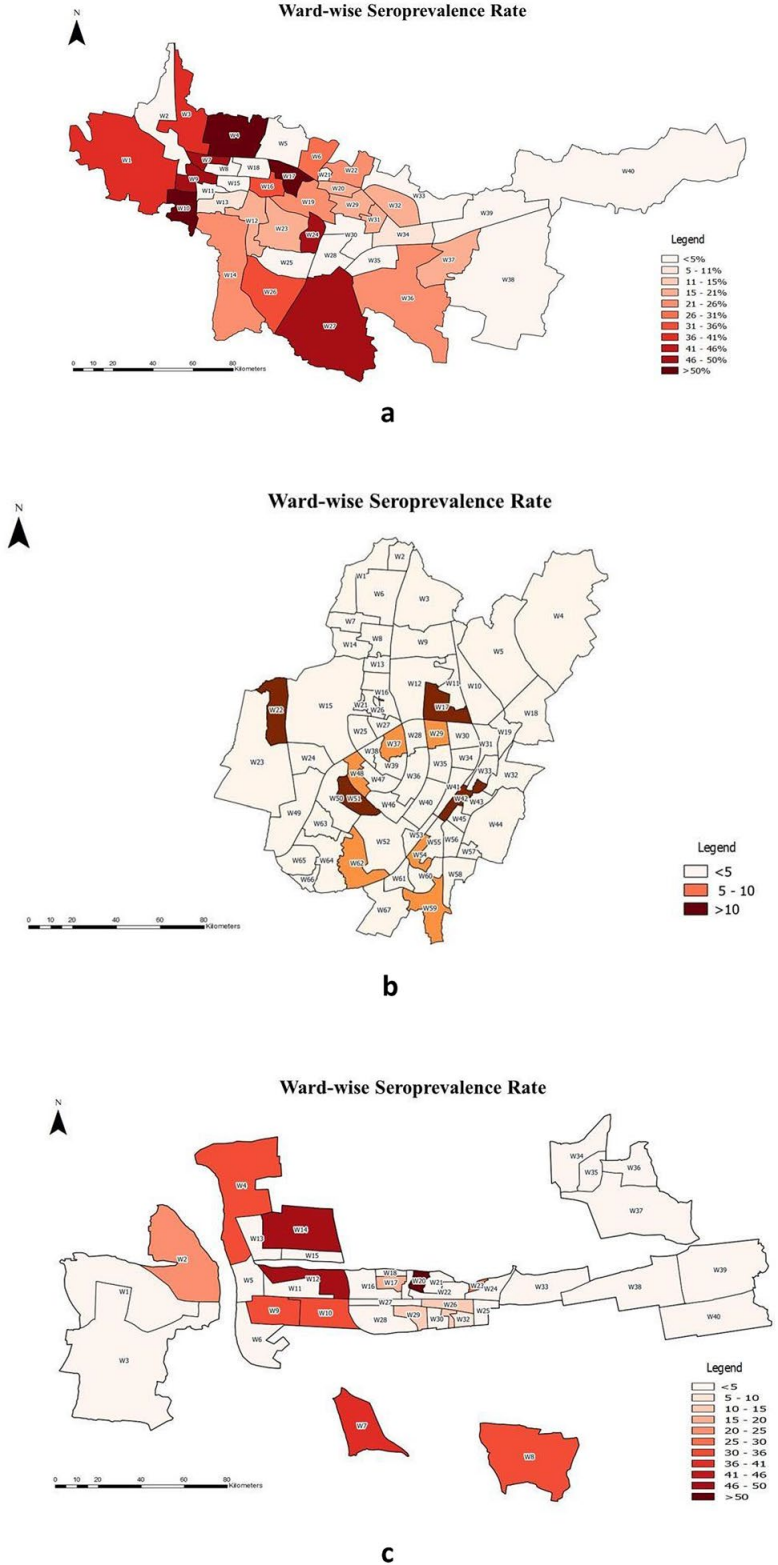
(**a**) Ward seroprevalence distribution of BAM. (**b**) Ward seroprevalence distribution of BBS. (**c**) Ward seroprevalence distribution of RKL.

The case-to-to-infection ratio on the date of serosurvey was 1:6.6 in BBS, 1:61 in BAM, and 1:29.8 in RKL. The heat maps for the geographical distribution of the seroprevalence across the three cities are given in Fig. 4.

## Discussion

This COVID-19 serosurvey involving more than 4000 participants from the three largest cities of Odisha found an overall seroprevalence of 20.78%, although there was wide variation in seroprevalence between the cities. The study included only the adult population. As educational institutions remain closed and lockdowns of various degrees have been imposed in the study setting since the pandemic began, the likelihood of adults being the source of household infection is high.

Although molecular tests are being used for the diagnosis of active symptomatic and asymptomatic cases of COVID-19, antibody-based tests can provide a more robust and comprehensive knowledge about the actual spread of infection in the community^17^. Seroprevalence studies on COVID-19 have been reported globally to assess the spread of infection either in the general population or focussing on certain high risk groups. Seroprevalence studies performed on health care workers across countries such as Germany, Belgium, United Kingdom, Malawi, and Italy, have reported wide variation in seropositivity ranging from 1.6% in Germany to 15.6% in Pakistan^18–23^. However, such studies on the population involved in high risk professions, although useful in informing trends of infection in such occupations, do not provide information about exposure at the population level and inferences possible are limited.

Globally, there have been few reports on seroprevalence against COVID-19 in the community. An early study on seroprevalence from Lombardy, Italy, involving 390 blood donors showed that 23% of the donors were positive for anti-COVID-19 neutralizing antibodies^24^. A community-based study from British Columbia in Canada, involving serial cross-sectional sampling, reported a seroprevalence of only 0.28% in March 2020, and 0.55% in May 2020^25^. Another community-based weekly serosurvey conducted in April 2020 in Geneva, Switzerland, reported a seroprevalence of 3.1%, 6.1%, and 9.7% during the first, second, and third week respectively, with a significantly higher seroprevalence in less than 50 years age group^26^. Such studies involving periodic sampling are extremely valuable in monitoring the spread of infection in certain areas and understanding the dynamics of community transmission as well as residual susceptibility^25^. In China, the seroprevalence in Guangzhou and Wuhan, the epicentre of the COVID-19 pandemic, was reported to be 0.6% and 2.1% respectively till April 2020^27^. During the same month, a study from Santa Clara County, California, reported a seroprevalence of 2.8% (95% CI 1.3–4.7%)^28^. These studies across countries indicate that the actual spread of COVID-19 infection in the community was much higher than that reported by the detection of active cases using molecular methods. Our study also presents evidence to support this case.

Few studies on the seroepidemiology of COVID-19 have been reported from Asia to date. A national serosurvey in India conducted during May–June, 2020, which included 28,000 individuals from 70 districts of 21 Indian states, reported the seroprevalence to be approximately 0.73% (95% CI 0.34–1.13)^29^. This indicated that the cumulative COVID-19 infection in India was approximately 6.46 million by the beginning of May 2020. Compared to our study, the low seroprevalence reported in the national serosurvey may be due to the difference in the study period (May–June in the national serosurvey compared to August in our study). While our study showed females to be more infected, the national serosurvey found males to have significantly higher seroprevalence. The infection to case ratio (ICR) in this national serosurvey varied between 81.6 and 130.1 with May 11 and May 3, 2020, as reference points for reported cases^29^. This is higher than that reported in our study (ranging from 6.6 in Bhubaneswar to 61 in Berhampur). The steady increase in testing and subsequent improvement in case identification may be the reason for this difference. Similar to our study, the national serosurvey also reported a higher seropositivity rate in occupations with a high risk of exposure to potentially infected persons^29^. Very few other countries in Asia have conducted serosurveys for COVID-19 in their general population. A recently reported community-based study from Karachi, Pakistan, has reported a seroprevalence of 8.7% (95% CI 5.1–13.1) and 15.1% (95% CI 9.4–21.7) in low and high transmission areas respectively, with no significant difference between males and females^30^. Similarly, the seroprevalence reported from two community clinics in Tokyo was 3.83% (95% CI 2.76–5.16)^31^. While others have reported a higher seroprevalence among the elderly, our study did not find any difference between the age groups with respect to their seropositivity. Thus, the evidence to date is showing significant regional as well as time-dependent variations in the findings of serosurveys to assess the exposure to SARS-CoV-2. Most serosurveys, including ours, have reported a large majority of infections to be asymptomatic. Among the symptomatic cases, the most common symptom was fever followed by cough and diarrhea as a symptom was strongly associated with seropositivity.

An interesting finding is the higher seropositivity in members of larger households, indicating the higher risk of household transmission among them. Correlations with the actually detected epidemic curves of the three cities show a trend where with higher seroprevalence, there is consistent relative flattening. Thus, the possibility of herd immunity being achieved at some point of time in the population cannot be ruled out. GIS analysis shows that wards detected with high seropositivity did not necessarily report more detected cases, implying gaps in testing in those regions.

Our study had a few limitations. The participants were only adults and the nonresponse rate were high (17.4%), and hence, the possibility of selection bias cannot be excluded. The non-response was higher among females probably due to cultural factors and higher individual apprehension towards blood sample collection. The study reported on the prevalence of antibodies against SARS-CoV-2 at a point in time. Follow up data on anti-SARS-CoV-2 antibodies in the same subjects will be required to understand the duration of immunity to natural infection as well as protection against reinfection. Serial cross-sectional serosurveys have been planned in the same population to address this issue and estimate the rate of spread of COVID-19 infection in Odisha.

To conclude, our study found a high seroprevalence against COVID-19 in urban Odisha and there seems to be a correlation between community seroprevalence and the so-called “flattening of the curve”. Future studies integrating seroprevalence data with sociocultural and other biological data will help us better understand the dynamics of COVID-19 transmission and the susceptibility to infection at the individual and community level. It will also help us understand the effectiveness of several steps undertaken by the state and central government such as social distancing, usage of masks, etc., in preventing the spread of COVID-19 infection in the community. However, we should be careful while interpreting the findings of a seroprevalence study. There is still no concrete data to support the fact that the presence of antibodies against COVID-19 is protective against reinfection. Moreover, seroprevalence studies should not be used to stigmatize any community or politicized to underestimate the efforts of any government in reducing the spread of infection in their respective countries.
